# A Case of Secondary Pulmonary Hypertension in a Patient With Atrial Septal Defect and Fetal Alcohol Syndrome

**DOI:** 10.7759/cureus.65611

**Published:** 2024-07-28

**Authors:** Maja Pirnat, Vesna Lesjak, David Šuran, Tina Lovrec Orthaber

**Affiliations:** 1 Department of Radiology, Maribor University Medical Centre, Maribor, SVN; 2 Department of Cardiology and Angiology, Maribor University Medical Centre, Maribor, SVN; 3 Department of Radiology, Community Healthcare Center Dr. Adolf Drolc Maribor, Maribor, SVN

**Keywords:** fetal alcohol spectrum disorders, prenatal alcohol exposure, cognitive impairment, secondary pulmonary hypertension, atrial septal defect (asd), fetal alcohol syndrome (fas)

## Abstract

We report a case of a 34-year-old man with fetal alcohol syndrome (FAS) presenting with dyspnea, cough, and hoarse voice. The patient was found to have severe pulmonary hypertension secondary to a large atrial septal defect (ASD). In this article, we discuss the challenges patients with FAS and other patients with cognitive impairments face that could explain the first diagnosis of such a large cardiac birth defect being made in the patient’s adulthood. Moreover, severe pulmonary hypertension due to ASD also presents a therapeutic dilemma, as shunt closure can lead to a worsening of the condition.

## Introduction

Congenital heart diseases are the most common birth defects as well as the leading cause of perinatal mortality [1]. Atrial septal defects (ASD) are amongst the more common congenital heart diseases, with a prevalence of 1.6 per 1,000 live births that have a 97% survival probability into adulthood [2]. In this article, we present a patient diagnosed with fetal alcohol syndrome (FAS) with ASD and severe pulmonary hypertension.

## Case presentation

A 34-year-old man presenting with dyspnea, cough, and hoarse voice was admitted to the Department of Cardiology of the Maribor University Medical Centre, Maribor, Slovenia due to suspected pulmonary hypertension.

Upon reviewing the medical history, it was found that the patient has known FAS with mild cognitive impairment. After losing his mother at the age of three and his alcoholic father at the age of eight, he grew up in foster care and received basic education at a school specializing in children with special needs. Currently, he works at a poultry farm. The patient’s medical history data was scarce, with no mention of any previous cardiac ultrasound (US) or other workup for a possible cardiac anomaly. Prior to hospitalization, he visited a pulmonology specialist in a smaller medical center, because he was suffering from dyspnea for two years. The specialist ordered a thoracic computed tomography (CT) scan. The scan was performed in native and portal venous phase, it demonstrated enlarged pulmonary arteries and a dilated right ventricle. Thus, pulmonary hypertension was suspected and he was referred to the Maribor University Medical Centre for further investigations.

**Figure 1:**
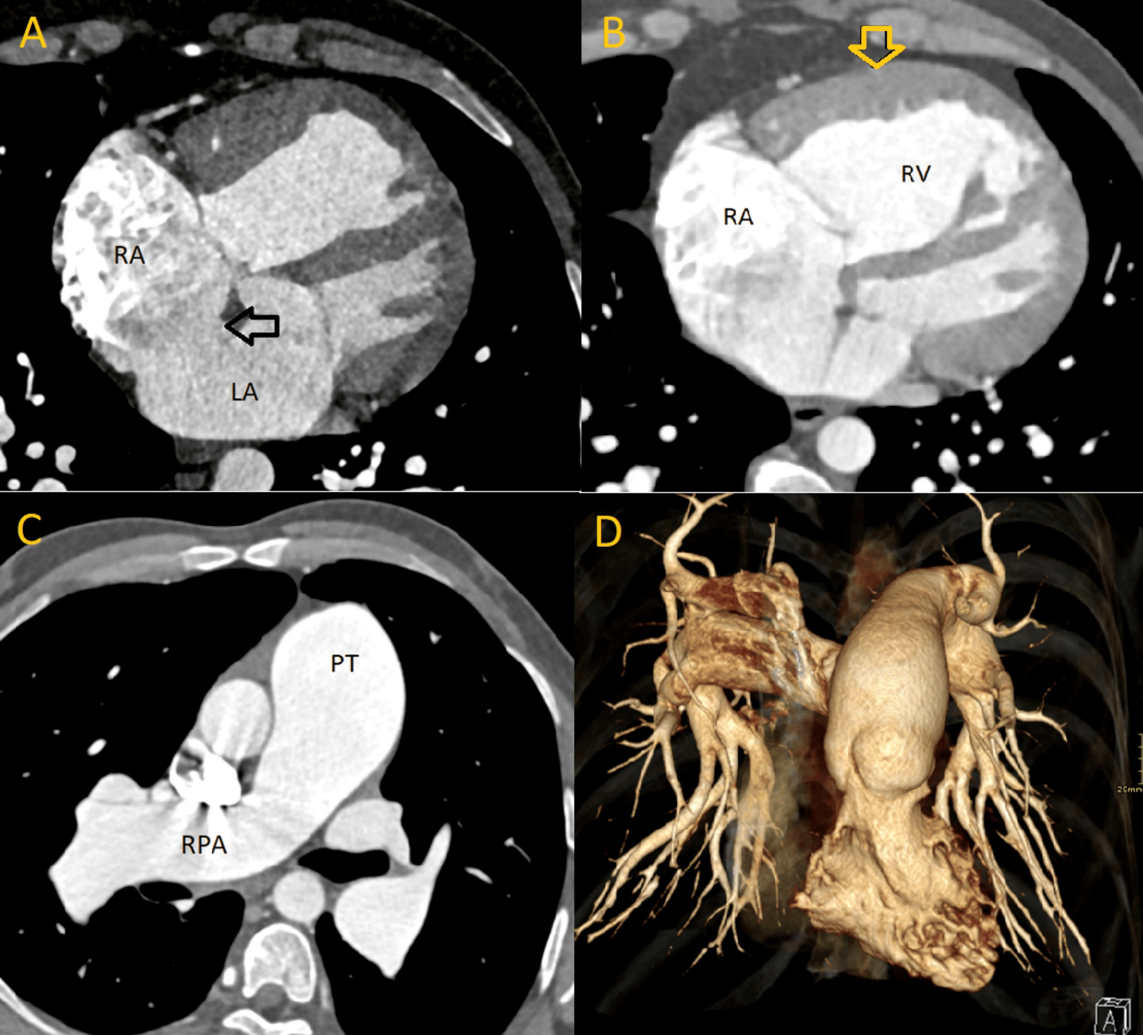
CT images of the heart and pulmonary arteries. A) long-axis view of the heart with ASD (black arrow); B) right heart chambers with thickened myocardium (yellow arrow); C) pulmonary truncus and right pulmonary artery; D) 3D reconstruction of the pulmonary arteries RA: right atrium; LA: left atrium; RV: right ventricle; PT: pulmonary truncus; RPA: right pulmonary artery

**Figure 2:**
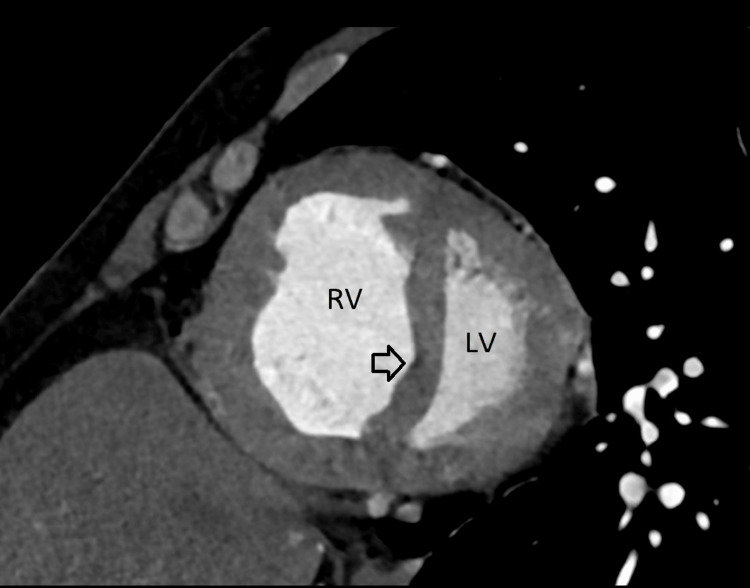
Bulging of the septum (arrow) into the left ventricle RV: right ventricle; LV: left ventricle

**Figure 3:**
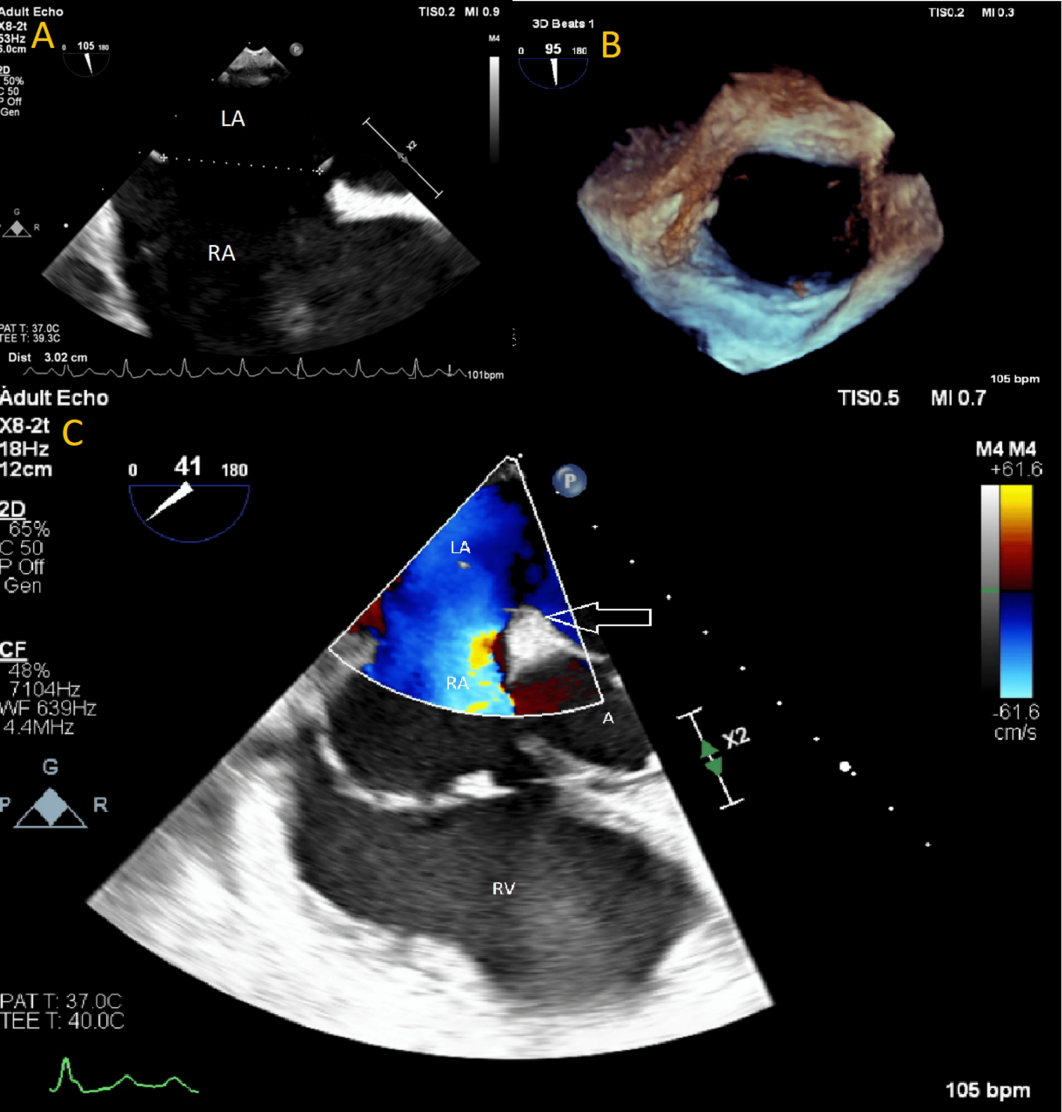
TEE images. A) ASD measuring 3 cm; B) 3D reconstruction of the ASD; C) TEE with color Doppler demonstrating left to right shunt LA: left atrium; RA: right atrium; RV: right ventricle; A: aorta; ASD: atrial septal defects; TEE: transesophageal echocardiogram

Cardiac US and CT angiography of the pulmonary arteries and heart were performed. The first cardiac US demonstrated severe pulmonary hypertension but did not mention a shunt between the atria. Only upon performing a CT angiography an ASD ostium secundum measuring 3 cm was documented (Figure 1A). On CT angiography the right heart chambers were enlarged with thickened myocardium of the right ventricle measuring 1 cm (Figure 1B), and bulging of the septum into the left ventricle (Figure 2). The central pulmonary arteries were also enlarged (Figure 1C). Pulmonary function tests demonstrated obstruction with a 50% reversibility. During hospitalization, the patient was treated for *Staphylococcus aureus* sepsis due to thrombophlebitis. Consequently, a transesophageal echocardiography (TEE) was performed to rule out endocarditis. The TEE showed an ASD secundum (Figure 3).

On invasive coronarography, the coronarogram and ventriculogram were normal; however, severe pre-capillary pulmonary hypertension was found with, a vascular resistance of 5 Wood units (WU), systemic vascular resistance of 7.1 WU, and a hemodynamically significant left to right shunt due to the ASD. Systolic pressure in the pulmonary artery was 75 mmHg, diastolic pressure was 37 mmHg, and mean pressure was 52 mmHg.

**Figure 4:**
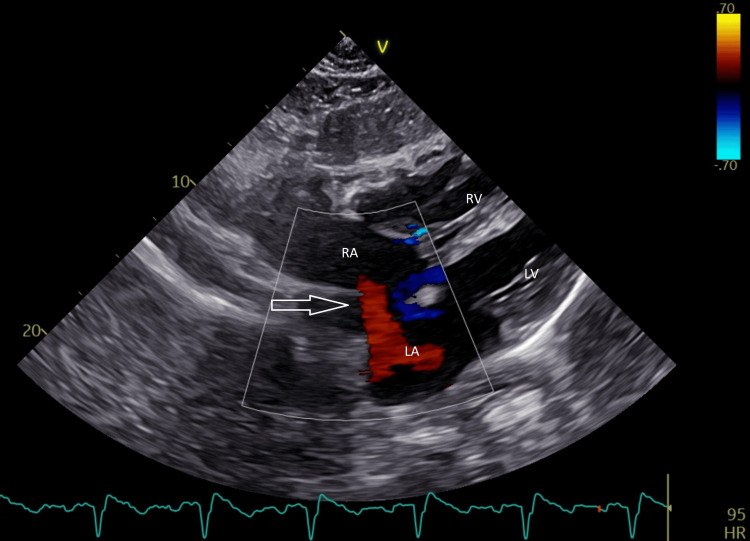
Transthoracic US with color Doppler demonstrating ASD (arrow) with bidirectional shunt LA: left atrium; RA: right atrium; RV: right ventricle; LV: left ventricle; ASD: atrial septal defects; US: ultrasound

The University Medical Centre Ljubljana was consulted regarding the possibility of surgical or endovascular shunt closure. It was decided that the patient would first be treated with pulmonary antihypertensive drugs while considering the possibility of performing a postponed surgical closure if the response to drug treatment is adequate. While awaiting the response to drug treatment, a follow-up transthoracic US was performed. The ASD shunt was found to be a mainly left-to-right bidirectional shunt (Figure 4) and the pulmonary hypertension was still found to be severe. A final conclusion on whether surgical closure will be performed after all, has yet to be reached even after six months since the initial evaluation for the possibility of closure.

## Discussion

According to the diagnostic criteria published in 1996 by the Institute of Medicine, disorders due to prenatal alcohol exposure (PAE) are termed fetal alcohol spectrum disorders (FASD). FASD is comprised of 4 types: FAS, partial FAS, alcohol-related neurodevelopmental disorder, and alcohol-related birth defect (ARBD) [3]. ARBD includes malformations such as ASD, ventricular septal defects, aberrant great vessels, conotruncal heart defects, as well as other defects of the skeletal system, and internal and sensory organs [4,5]. While our patient has a known history of FAS with mild cognitive impairment, the ASD, however, was a new finding.

ASDs are defined as defects of the interatrial septum with direct communication between both atria or defects that physiologically behave like ASDs. Ostium primum ASD, endocardial cushion defects, ostium secundum ASD represent the first type, and sinus venosus ASD and unroofed coronary sinus represent the second type [6]. In this case, an ostium secundum type ASD was found, which is the most prevalent type of ASD. They form either due to excessive apoptosis of the cephalic portion of the septum primum or the incomplete development of the septum secundum. ASDs ostium secundum can be multiple or associated with fenestrations. On echocardiography, they are usually located in the central portion of the interatrial septum. In cases with poor acoustic windows on transthoracic US, a transesophageal US and real-time three-dimensional techniques may provide more information on size, shape, and number of defects. On cardiac CT the defect can be seen in the fossa ovalis region. Distinguishing between an ostium secundum and patent foramen ovale (PFO) can be challenging; however, a PFO will usually have a contrasting flow to either the roof of the left or the floor of the right atrium, while the flow tends to be more horizontal in an ostium secundum. Besides contributing to the assessment of the morphology of the ASD, cardiac CT also aids in the evaluation of other structures, such as the right chambers and pulmonary arteries [7].

In the presented case the ASD was not mentioned in the first cardiac US examination, it was diagnosed only upon performing a cardiac CT. It is important to note, that while echocardiography is usually the method of choice in diagnosing ASDs, CT methods have comparable diagnostic accuracy. In a study by Li et al. in 2017, the diagnostic accuracy for echocardiography was 98.40% and for 64-multislice CT was 96.20% [8]. Moreover, echocardiography is heavily operator-dependent and requires intensive training and highly qualified personnel [9]. On the other hand, the role of cardiac CT has been growing, especially in cases where cardiac US is limited, as it offers a high spatial resolution [7].

Our patient was first diagnosed with ASD at the age of 34 when he already presented with severe pulmonary hypertension. Upon review of medical history, no previous cardiac US was mentioned. We also noted that his medical history was rather scarce, considering the fact that he was diagnosed with FAS and mild cognitive impairment. We believe that many factors contributed to such a late diagnosis of ASD. As a child, our patient grew up in an unstable environment with alcoholic parents and later moved to foster care. The continuity of physical and mental health care is often compromised in foster care as foster parents only have limited information on the child’s medical history and biological family [10]. Furthermore, studies suggest that caregivers of children with mild cognitive impairment hold the belief that these children can overreact to pain in comparison to children without impairment. It is also reported that caregivers see a disconnect between the children’s pain sensation and reaction to pain in these children [11]. Research also shows that cognitive impairment affects a physician’s decision-making in the treatment of such patients. Sometimes the physician's assumptions are not evidence-based, this could explain why many patients with mild cognitive impairment receive fewer tests and/or less effective treatments [12].

The connection between ASDs and PAE has been questioned in recent years. According to the meta-analysis conducted by Zhang et al. in 2020, no statistically significant association has been found between ASDs and parental alcohol consumption [1]. A previous meta-analysis by Yang et al. reported similar findings, where they also noted that most studies that did find a significant association were published before the year 2000 [13]. An Australian study in 2013 did report an association between ASD and any kind of maternal alcohol diagnosis, but they also stated that none of the studied ARBDs were 100% attributable to PAE [14]. Other authors also emphasized the limitation of many coinciding factors that could also lead to the development of a congenital heart disease such as maternal smoking, other substance abuse, a poor diet, genetic causes, etc. [15]. Another limitation noted in the research was that many studies did not report the risk for specific congenital heart diseases [1].

Our patient presented with pre-capillary and capillary pulmonary hypertension. Pulmonary arterial hypertension (PAH) associated with ASD may present with three different clinical phenotypes. The first category consists of adults with mild PAH and a large shunt. The second includes adults also with a large defect, but severe pulmonary vascular disease that is irreversible and accompanied by shunt reversal and chronic cyanosis. The third category presents with a small defect with severe and disproportionate PAH, in this case, the ASD is usually more of a coincidental finding and not the cause of increased pulmonary artery pressure. These different types require different treatment approaches. While patients with a large shunt and only mild PAH may safely undergo ASD closure, in cases with a large shunt, and severe PAH with shunt reversal, closure would be counterproductive and detrimental. Pulmonary vasodilators would be the treatment of choice in the latter group. It is, however, often challenging to choose the best approach, as people rarely fall neatly into these categories. These patients require a multidimensional case-by-case approach, as pre-tricuspid shunts such as ASDs with severe pulmonary hypertension are far more challenging from the viewpoint of operability compared to post-tricuspid shunts. That is due to the fact that in contrast to post-tricuspid shunts, which are mainly systolic or continuous, an ASD shunt is mainly diastolic and can persist as a left-to-right shunt despite elevated pulmonary artery pressure and vascular resistance, becoming bidirectional only once right ventricular diastolic pressure is elevated, meaning that the absence of desaturation at rest or exertion cannot exclude irreversible PAH [16]. Closure can be performed surgically or percutaneously, although, in recent years percutaneous closure has become the treatment of choice with an incidence of severe complications in less than 1% [17]. According to the ESC Guidelines for the management of grown-up congenital heart disease, the indication for ASD closure is the presence of a significant shunt with concomitant right ventricular overload in the absence of severely increased pulmonary vascular resistance (>5 WU). ASD closure is contraindicated in patients with Eisenmenger physiology, patients with PAH and pulmonary vascular resistance >5 WU despite PAH treatment, or desaturation on exertion [18]. In our case the patient had a vascular resistance of 5 WU and a left-to-right shunt due to the ASD at the beginning of the evaluation, thus being a borderline case, falling into the category where a shunt closure is still a feasible option. It was, however, decided that the patient would initially undergo pharmacological treatment with the possibility of postponed surgical closure.

## Conclusions

In this case report we wish to highlight the following learning points. First, the role of cardiac CT has been on the rise in recent years as demonstrated in this case with CT being the first method to diagnose ASD. Second, the perception and treatment of patients with cognitive impairment are affected by the beliefs of caregivers and physicians, which can lead to poorer health outcomes. Although the connection between ASDs and PAE has been questioned in recent years, further research is needed to determine the association between the two. PAH in the setting of ASD presents a therapeutic dilemma, as shunt closure may worsen the condition in certain patient groups.
